# Using Entropy to Measure Religious Pluralism

**DOI:** 10.3390/e27010047

**Published:** 2025-01-09

**Authors:** George Sturm

**Affiliations:** Radio Logos, 7301 Pogradec, Albania; geosturm01@aol.com

**Keywords:** religious pluralism, measuring pluralism, diversity, entropy, cluster analysis

## Abstract

This paper discusses a unique and revolutionary method that quantitatively evaluates the programming of a media outlet regarding religious pluralism. Using a “program appeal” score and entropy measures from information theory, the broadcast operator is able to determine if governmental compliance is being met and whether certain programs are problematic. The theoretical foundation of this tool is presented and illustrated using real-life data.

## 1. Introduction

Radio Logos is the largest regional FM radio network in the European country of Albania. It currently consists of seven transmission sites which cover a wide geographical area of the country. The governmental agency over Radio Logos is the Albanian Audiovisual Media Authority (AMA), which is responsible for licensing radio and television stations and ensuring their compliance with the broadcasting laws of the country. One aspect of these laws is that the content of our programs must clearly express general pluralism and impartiality of information. This includes religious pluralism.

Unfortunately, the exact meaning of pluralism is not well defined in the academic literature. Sometimes the expression “diversity” is used as a synonym (which we use in this paper), but without a precise definition of these terms, especially with regard to how to quantitatively measure pluralism, discussions on this matter often degenerate into futile exchanges of subjective opinions.

This lack of clarity also includes what broadcasting programs should be included in a discussion on pluralism. Although the reader will discover that our study encompasses all programs transmitted during a typical broadcast day, it must be pointed out that not all agree with this viewpoint. For example, Ofcom (The Office of Communications, the government-approved regulatory and competition authority for the broadcasting, telecommunications, and postal industries of the United Kingdom) says “*We recommended that the scope of any plurality review should be limited to news and current affairs, but that these genres should be considered across television, radio, the press and online*” [1]. The BBC also expressed a similar opinion [2], stating, “*The BBC concurred that news and current affairs are the most important genres in terms of driving the news agenda and developing public debate, and so therefore should be the primary genres to be considered in any plurality policy*.”

There are even some who express concern that the concept of pluralism is not only ambiguous but lacks any universally recognized standards for evaluating media performance and quality [3].

Perhaps the only area of consensus is that governmental agencies like AMA should aim to promote a diverse range of media outlets (e.g., radio and television stations) in order to bring about pluralism; that is, increased diversity [1,4,5]. The idea is that each media outlet will have its own unique programming characteristics and that, together, they will create a pluralistic voice for the public. Thus, an individual will have the opportunity to choose from a diverse and pluralistic set of media choices.

It also must be noted that the academic literature is sparse when it comes to correlating pluralism with an individual media outlet and its programming. How can such a correlation be measured quantitatively? This paper attempts to solve this problem by presenting a self-evaluation tool based upon program appeal scores, entropy measures from information theory, and Cluster Analysis. This tool will help the operator identify pluralism compliance status and areas of programming concern. Real-life data will be used to illustrate its usage.

## 2. Methods

Although the following discussion will focus on religious pluralism, the methods presented can be applied to other areas of concern (e.g., gender and racial diversity).

First, we use the results from the 2023 Albanian census [6], which shows the general religious groupings with their respective population percentages. For example, Muslims make up roughly 51% of the population, Christians 16%, Atheists 17%, and Non-declared (ND) 16%. However, it must be understood that these categories are not rigid and fixed; that is, there is a lot of cross-over when it comes to people’s religion. It is no secret that, practically speaking, religious observance in Albania is generally lax, and few Albanians consider religion to be a dominant factor in their lives. When asked about religion, people generally refer to their family’s historical religious legacy and not to their own choice of faith. It is also very common that people will say that they are half of one religion and half of another. Nevertheless, we will use the results of the 2023 census.

The next step is to analyze each program that is being broadcast by Radio Logos to determine the likelihood that this program would appeal to a person of a particular religious grouping. We call this “program appeal”.

To better understand this concept of “program appeal”, for comparison purposes, we first create some illustrative and representative programs along with their appeal numbers in the following table. These “appeal” numbers are values between 0 and 1 (a score of 0.5 would mean that the program appeals to 50% of the listeners). See Table 1 and Table 2 for details.

Since the evaluation method of this paper is presented as a self-evaluation tool, it must be understood that these assigned values are not based upon any sophisticated scientific data analysis. Rather, they were thoughtfully chosen based upon what we believed was a reasonable level of program acceptance by the various religious groups.

Using these illustrative programs and appeal numbers, we calculate four statistics that measure acceptance, pluralism and diversity. The first is a simple weighted average and the other three are based on information theory.

### 2.1. Overall Appeal

This is the weighted average of the religious grouping percentage with the likelihood that the particular program appeals to that group.

$$\begin{array}{c} overall=0.51*prob\left(appeal\;given\;Muslim\right)+0.16*prob\left(appeal\;given\;Christian\right)+0.17\\ {}*prob\left(appeal\;given\;Atheist\right)+0.16*prob\left(appeal\;given\;nondeclared\right) \end{array}$$

For example, for the offensive program (OF_ALL) we have,

overall=0.51∗0.10+0.16∗0.10+0.17∗1.0+0.16∗0.10=0.253

### 2.2. Normalized Shannon Entropy

The expression “entropy” was introduced into the field of communications by Claude Shannon, an American mathematician and engineer. As a result, it is often called Shannon entropy, since he was the “the father of information theory”. The concept of entropy is widely used in many scientific fields and is a measure of the diversity of a communication system [7]. Its formula is as follows:

$$Ent=-\sum_{i=1}^{4}p_{i}{log}_{2}\left(p_{i}\right)/{log}_{2}(n)$$

where

n=is the number of religious groups (in our case n=4)

and

$$p_{1}=0.51*prob\left(appeal\;given\;Muslim\right)/overall$$

$$p_{2}=0.16*prob\left(appeal\;given\;Christian\right)/overall$$

$$p_{3}=0.17*prob\left(appeal\;given\;Atheist\right)/overall$$

$$p_{4}=0.16*prob\left(appeal\;given\;nondeclared\right)/overall$$

which means that

$$\sum_{i=1}^{4}p_{i}=1$$

The maximum value of Ent is 1.0. For example, for OF_ALL, the calculations are as follows:

$$p_{1}=0.51*\frac{0.1}{0.253}=0.2016$$

$$p_{2}=0.16*\frac{0.1}{0.253}=0.0632$$

$$p_{3}=0.17*\frac{1.0}{0.253}=0.6719$$

$$p_{4}=0.16*\frac{0.1}{0.253}=0.0632$$

$$Ent=-\left(0.2016{log}_{2}\left(0.2016\right)+0.0632{log}_{2}\left(0.0632\right)+0.6719{log}_{2}\left(0.6719\right)+0.0632{log}_{2}\left(0.0632\right)\right)/{log}_{2}(4)=0.6775\;$$

### 2.3. Reflective Diversity (RD)

Reflective diversity [8] is a measure against which existing population preferences are proportionally represented in the media programming. We are applying this statistic to measure reflective religious diversity. Its formula is

$$RD=1-\sum_{i=1}^{4}abs\left(p_{i}-{norm}_{i}\right)/2$$

where

$${norm}_{1}=0.51$$

$${norm}_{2}=0.16$$

$${norm}_{3}=0.17$$

$${norm}_{4}=0.16$$

For example, for OF_ALL, the calculations are as follows:

$$RD=1-\left(abs\left(0.2016-0.51\right)+abs\left(0.0632-0.16\right)+abs\left(0.6719-0.17\right)+abs\left(0.0632-0.16\right)\right)/2=0.498$$

### 2.4. Open Diversity (OD)

This statistic [8] measures how diverse preferences and opinions are uniformly represented in the media. We are applying this statistic to measure open religious diversity. Its formula is

$$OD=1-\sum_{i=1}^{n}abs\left(p_{i}-1/n\right)/2$$

where n = 4.

For example, for OF_ALL, the calculations are as follows:

$$RD=1-\left(abs\left(0.2016-0.25\right)+abs\left(0.0632-0.25\right)+abs\left(0.6719-0.25\right)+abs\left(0.0632-0.25\right)\right)/2=0.5781$$

Using these statistics with our illustrative programs and appeal scores, we have the following results (Table 3).

The question, therefore, is as follows: How do we collectively use these data to evaluate our programming regarding religious diversity? The answer is Cluster Analysis.

Cluster Analysis is a classical statistical technique that helps the researcher “reduce” or “simplify” data into similar clusters. In our situation, Cluster Analysis will group the programs into similar clusters based upon the variables Overall, Entropy, RD and OD. Applying this technique to our data, a Cluster Dendrogram is produced, which is a graphical method used to assess which programs are similar.

**Figure 1:**
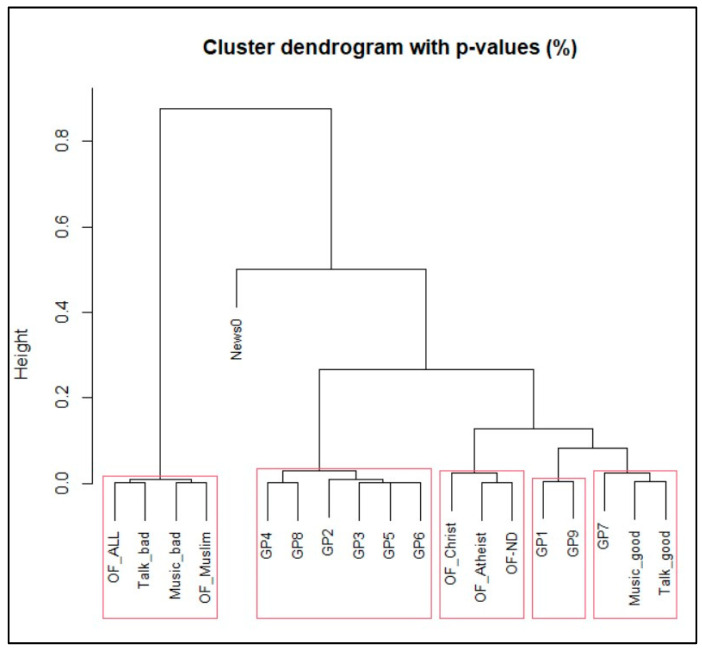
Dendrogram for the representative programs.

Using the pvclust method [9] from the R software package version 4.2.1. [10] and applying this to our illustrative and representative data, we obtain the following Dendrogram (see Figure 1) with several rectangles that highlight “significant” clusters. Internally, the package uses the AU (Approximately Unbiased) probability value to construct these clusters. The idea is that if the AU value is greater than or equal to 99, then we can group programs into a single cluster.

The Dendrogram shows that the illustrative and representative programs can be grouped into six clusters (see Table 4).

This clustering makes perfect sense in that programs that are offensive to some degree or lack religious diversity are grouped into separate clusters. Other “good” programs are likewise grouped into their own clusters. News is a cluster by itself.

Let us now introduce (see Table 5) the programs of Radio Logos (from a typical weekday). The appeal scores were based upon historical listener feedback.

It must be noted that we did not conduct an in-depth professional survey to produce these values, since no radio station has the time, resources and money to facilitate such a thing. Furthermore, our method is meant to be a self-evaluation tool. Thus, we carefully based our numbers upon almost 20 years of listener feedback, talking to people on the street, and general conversations with people from all walks of life and religious backgrounds. In other words, since we know our programs well and are familiar with what people think of them, we were able to assign them reasonable appeal scores.

Using the above scores, we calculate the statistics Overall, Entropy, RD, and OD. These are listed in Table 6.

**Figure 2:**
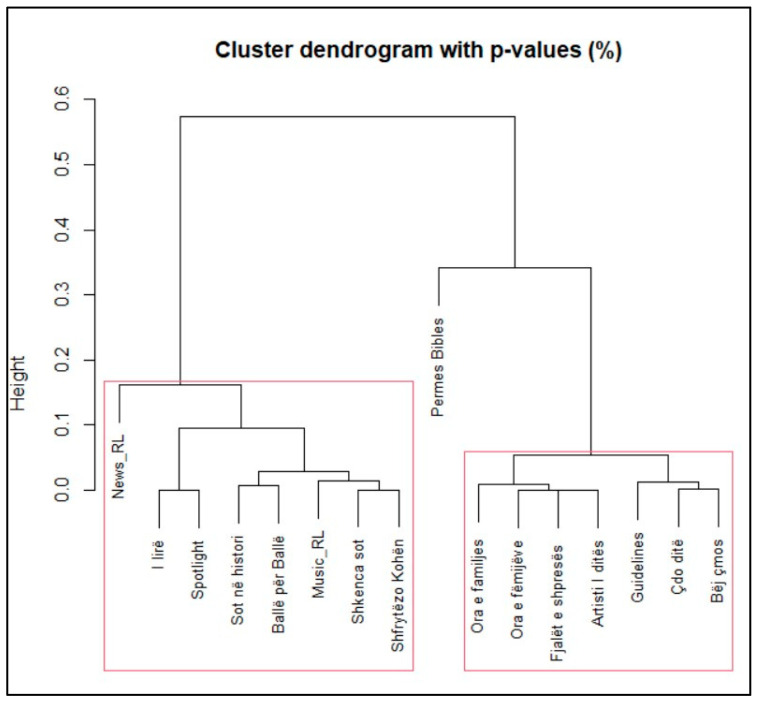
Dendrogram for Radio Logos programs.

Next, we perform Cluster Analysis on the data. The analysis extracts three main clusters (see Figure 2). The obvious highlight is a program called “Permes Bibles” which stands alone as a cluster.

**Figure 3:**
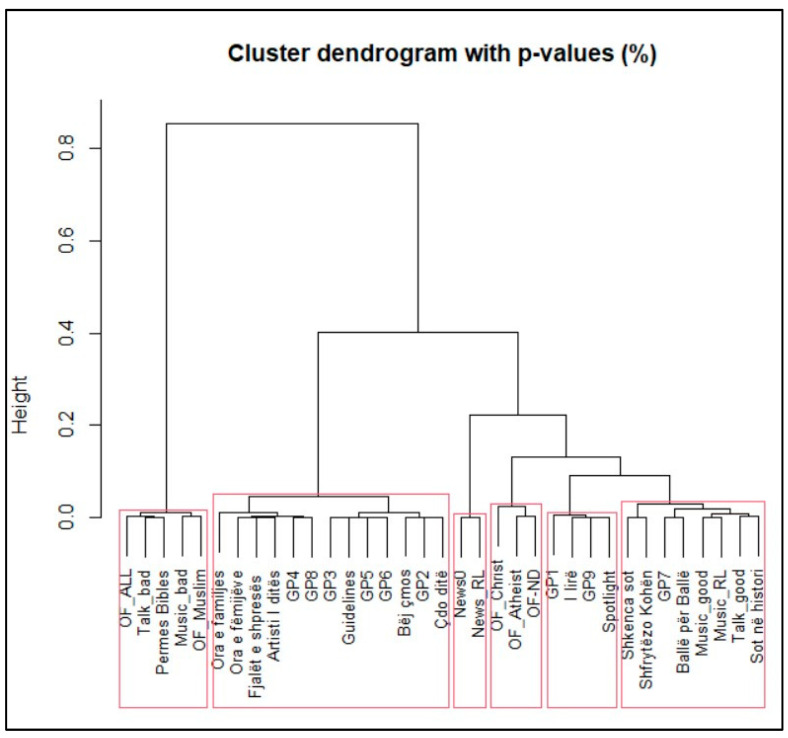
Dendrogram for the combined programs.

Finally, we combine the data from illustrative and representative programs with the programs of Radio Logos and use Cluster Analysis. This produces six significant clusters (see Figure 3).

## 3. Results

The results show that no Radio Logos program falls into the cluster which is offensive toward Christians, Atheists, and Non-declared people. Radio Logos news is comparable with the ideal illustrative news category. Except for one program, all other Radio Logos programs fall into clusters with other general programs. The only exception is “Permes Bibles” which, unfortunately, falls into the cluster with a number of potentially offensive programs. Therefore, this program needs to be considered as a possible program that is not sufficiently religiously diverse.

A further examination of the data revealed that the programming of Radio Logos is not very appealing to Atheists. Specifically, the combined appeal scores of Muslims, Christians, and ND was 0.791, compared to the score of 0.369 for Atheists. Without question, no radio station can be appealing to everyone. Nevertheless, this presents a challenge for us at Radio Logos to be creative in our programming towards Atheists.

Overall, except for one possible program, this self-evaluation tool shows that Radio Logos is doing a tremendous job of maintaining pluralism and diversity.

## 4. Conclusions

No media outlet should ever be satisfied with the status quo. The future always brings challenges in the form of a changing listener audience. Therefore, we must be diligent in monitoring our programs, refreshing our playlists, and creating new programs to ensure that they satisfy our goal of pluralism and diversity.

The self-evaluation tool presented in this paper is one way to achieve this. It is easy to understand and simple to implement. If used on a regular basis, it will provide the broadcast operator with valuable information that will help keep their station appealing to all religious groups, help them to remain pluralistic in the eyes of the government, and help the operator to maintain a competitive market advantage over their rivals.

Finally, further research needs to be conducted with regard to identifying and using additional diversity measures outside of the entropy realm. Investigations need to be made into using more innovative approaches to measuring diversity, perhaps by combining elements from existing methods or adapting them to better suit the specific context of the study.

## Figures and Tables

**Table 1:** Representative programs.

News0	A news program (News0) would be very generic regarding its religious content and would have a universal appeal, as listed in the table. One hundred percent of Muslims would have no objection, and so on.
OF_ALL	An offensive program (OF_ALL), one that is filled with cursing against God, denouncing of politicians and sexually inappropriate language, would only appeal to 10% of Muslims but be liked by Atheists, and so on.
Music_bad	Music that is highly immoral and represented by immoral people might be liked more by non-religious people than anyone else.
Talk_bad	A talk show filled with cursing, immoral talk and so on, which might be liked more by non-religious people than anyone else.
OF_Muslim	A program that a Muslim would not find appealing. A similar concept is for OF_Christ, OF_Atheist, and OF-ND (Non-declared)
GP1 through GP9	GP means General Program. We listed nine varieties, none of which were particularly offensive, but which were liked more by some groups and less by others.

**Table 2:** The appeal scores of the representative programs.

	Muslim 51%	Christian 16%	Atheists 17%	Non-Declared 16%
News0	1.0	1.0	1.0	1.0
OF_ALL	0.1	0.1	1.0	0.1
Music_bad	0.1	0.1	0.9	0.5
Music_good	0.9	0.9	0.2	0.5
Talk_bad	0.2	0.2	0.9	0.5
Talk_good	0.9	0.9	0.5	0.8
OF_Muslim	0.1	0.7	0.5	0.4
OF_Christ	0.8	0.1	0.5	0.4
OF_Atheist	0.8	0.8	0.0	0.5
OF-ND	0.8	0.8	0.5	0.0
GP1	0.8	0.8	0.8	0.8
GP2	0.7	0.9	0.1	0.5
GP3	0.7	0.8	0.4	0.5
GP4	0.6	0.7	0.3	0.6
GP5	0.7	0.9	0.4	0.6
GP6	0.7	0.8	0.5	0.7
GP7	0.9	1.0	0.9	0.9
GP8	0.6	0.9	0.3	0.6
GP9	0.8	0.9	0.8	0.8

**Table 3:** Overall appeal and entropy measurements.

	Muslim 51%	Christian 16%	Atheists 17%	Non-Declared 16%	Overall	Ent	RD	OD
Lajmet0	1.0	1.0	1.0	1.0	1.00	0.89	1.00	0.74
OF_ALL	0.1	0.1	1.0	0.1	0.25	0.68	0.50	0.58
Music_bad	0.1	0.1	0.9	0.5	0.30	0.83	0.55	0.72
Music_good	0.9	0.9	0.2	0.5	0.72	0.72	0.83	0.61
Talk_bad	0.2	0.2	0.9	0.5	0.37	0.91	0.70	0.81
Talk_good	0.9	0.9	0.5	0.8	0.82	0.83	0.93	0.69
OF_Muslim	0.1	0.7	0.5	0.4	0.31	0.97	0.65	0.87
OF_Christ	0.8	0.1	0.5	0.4	0.57	0.63	0.80	0.54
OF_Atheist	0.8	0.8	0.0	0.5	0.62	0.62	0.80	0.59
OF-ND	0.8	0.8	0.5	0.0	0.62	0.63	0.81	0.59
GP1	0.8	0.8	0.8	0.8	0.80	0.89	1.00	0.74
GP2	0.7	0.9	0.1	0.5	0.60	0.74	0.83	0.65
GP3	0.7	0.8	0.4	0.5	0.63	0.83	0.90	0.69
GP4	0.6	0.7	0.3	0.6	0.56	0.84	0.92	0.71
GP5	0.7	0.9	0.4	0.6	0.66	0.85	0.92	0.71
GP6	0.7	0.8	0.5	0.7	0.68	0.87	0.95	0.73
GP7	0.9	1.0	0.9	0.9	0.92	0.90	0.99	0.75
GP8	0.6	0.9	0.3	0.6	0.60	0.86	0.92	0.74
GP9	0.8	0.9	0.8	0.8	0.82	0.90	0.98	0.75

**Table 4:** Clusters of the representative programs.

Cluster 1	OF_ALL, Talk_bad, Music_bad, OF_Muslim.
Cluster 2	GP2, GP3, GP4, GP5, GP6, GP8
Cluster 3	OF_Christ, OF_Atheist, OF_ND
Cluster 4	GP1 and GP9
Cluster 5	GP7, Music_good, Talk_good
Cluster 6	News0 (news program)

**Table 5:** Programs of Radio Logos.

Program	Muslim 51%	Christian 16%	Atheists 17%	Non-Declared 16%	Short Description
News_RL	1.0	1.0	1.0	1.0	A general news program
Music_RL	0.9	1.0	0.2	0.5	We are famous for having good music
Shkenca_sot	0.9	0.9	0.1	0.8	Interesting to most, but not to most Atheists, since we often use science to prove that we did not come from monkeys.
Shfrytëzo_Kohën	0.9	1.0	0.1	0.8	A general program on how to effectively use your time
Ora_familjes	0.5	0.9	0.2	0.5	A program that has addresses a wide variety of topics—book readings, biographies, family, etc.
Sot_histori	0.9	1.0	0.6	0.9	A general informative program
I_lirë	0.8	1.0	0.5	0.9	A radio drama that gives hope to those lost in alcoholism and drugs
Çdo_ditë	0.7	0.9	0.1	0.5	Motivational thoughts for each day
Bëj_çmos	0.7	0.8	0.1	0.5	Motivational thoughts for each day
Fjalët_shpresës	0.6	1.0	0.3	0.6	A program that gives hope and covers a wide range of topics
Guidelines	0.7	1.0	0.3	0.6	Motivational thoughts for women and men
Artisti_ditës	0.6	1.0	0.3	0.6	A program with good music from a single artist
Ballë_për_Ballë	0.9	1.0	0.9	0.9	Interview program with people from many difficult backgrounds, economic statuses, and religious backgrounds.
Ora_fëmijëve	0.6	1.0	0.3	0.6	A well-received children’s program with music
Spotlight	0.8	0.9	0.8	0.8	A program for learning English that talks about interesting people, places and events
Permes_Bibles	0.2	0.9	0.1	0.5	A fairly religious-oriented program

**Table 6:** Radio Logos overall appeal and entropy measurements.

Program	Muslim 51%	Christian 16%	Atheists 17%	Non-Declared 16%	Overall	Ent	RD	OD
News_RL	1.0	1.0	1.0	1.0	1.00	0.89	1.00	0.74
Music_RL	0.9	1.0	0.2	0.5	0.73	0.73	0.83	0.62
Shkenca_sot	0.9	0.9	0.1	0.8	0.75	0.72	0.85	0.64
Shfrytëzo_Kohën	0.9	1.0	0.1	0.8	0.76	0.73	0.85	0.65
Ora_familjes	0.5	0.9	0.2	0.5	0.51	0.85	0.88	0.72
Sot_histori	0.9	1.0	0.6	0.9	0.86	0.86	0.95	0.72
I_lirë	0.8	1.0	0.5	0.9	0.80	0.87	0.94	0.74
Çdo_ditë	0.7	0.9	0.1	0.5	0.60	0.74	0.83	0.65
Bëj_çmos	0.7	0.8	0.1	0.5	0.58	0.73	0.84	0.64
Fjalët_shpresës	0.6	1.0	0.3	0.6	0.61	0.86	0.90	0.74
Guidelines	0.7	1.0	0.3	0.6	0.66	0.83	0.89	0.71
Artisti_ditës	0.6	1.0	0.3	0.6	0.61	0.86	0.90	0.74
Ballë_për_Ballë	0.9	1.0	0.9	0.9	0.92	0.90	0.99	0.75
Ora_fëmijëve	0.6	1.0	0.3	0.6	0.61	0.86	0.90	0.74
Spotlight	0.8	0.9	0.8	0.8	0.82	0.90	0.98	0.75
Permes_Bibles	0.2	0.9	0.1	0.5	0.34	0.88	0.67	0.78

## Data Availability

Data is contained within the article.
